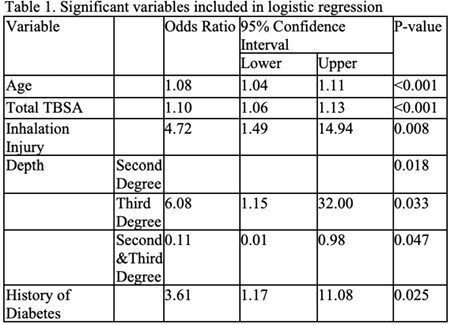# 504 Revised Baux Score Identifies a New Risk Factor for Mortality: History of Diabetes

**DOI:** 10.1093/jbcr/irae036.139

**Published:** 2024-04-17

**Authors:** Timothy Nehila, Marian Mikhael, Shreya Arora, Rithvic Jupudi, Jaynie X Criscione, Nicole K Le, Kristen Whalen, Kristina Buller, Jared Troy, Jake Laun

**Affiliations:** Morsani College of Medicine USF, Tampa, FL; USF Health Morsani College of Medicine, Tampa, FL; University of South Florida, Tampa, FL; Morsani College of Medicine USF, Tampa, FL; USF Health Morsani College of Medicine, Tampa, FL; University of South Florida, Tampa, FL; Morsani College of Medicine USF, Tampa, FL; USF Health Morsani College of Medicine, Tampa, FL; University of South Florida, Tampa, FL; Morsani College of Medicine USF, Tampa, FL; USF Health Morsani College of Medicine, Tampa, FL; University of South Florida, Tampa, FL; Morsani College of Medicine USF, Tampa, FL; USF Health Morsani College of Medicine, Tampa, FL; University of South Florida, Tampa, FL; Morsani College of Medicine USF, Tampa, FL; USF Health Morsani College of Medicine, Tampa, FL; University of South Florida, Tampa, FL; Morsani College of Medicine USF, Tampa, FL; USF Health Morsani College of Medicine, Tampa, FL; University of South Florida, Tampa, FL; Morsani College of Medicine USF, Tampa, FL; USF Health Morsani College of Medicine, Tampa, FL; University of South Florida, Tampa, FL; Morsani College of Medicine USF, Tampa, FL; USF Health Morsani College of Medicine, Tampa, FL; University of South Florida, Tampa, FL; Morsani College of Medicine USF, Tampa, FL; USF Health Morsani College of Medicine, Tampa, FL; University of South Florida, Tampa, FL

## Abstract

**Introduction:**

Mortality following burn injury is influenced by many objective factors. Over the past several decades, numerous predictive formulas have been developed to estimate the probability of death from burn injury. Despite the preponderance of models, there are relatively few widely accepted objective measures found to impact mortality in burn patients. These factors include sex, age, burn depth, TBSA, and presence of inhalation injury. In this study, we retrospectively analyzed mortality in the burn patients at our level one trauma center to identify prognostic factors.

**Methods:**

A retrospective chart review was performed on all patients between 2015-2020 over the age of 18 that presented to our trauma center for burns. Significant risk factors for the prediction of mortality based on a set of objective variables were identified using a stepwise forward logistic-regression analysis.

**Results:**

Of the 963 patients (mean [±SD] age, 47±17; mean TBSA, 9±13), 96% lived to discharge. The identified risk factors for death were increased age (OR 1.08, 95% CI 1.04-1.11, p< 0.001), increased TBSA (OR 1.10, 95% CI 1.06-1.13, p< 0.001), the presence of inhalation injury (OR 4.72, 95% CI 1.49-14.94, p=0.008), third degree burn depth (OR 6.09, 95% CI 1.15-32.00, p=0.033), and having diabetes (OR 3.61, 95% CI 1.17-11.08, p=0.025 (Table 1). The probability of death from patients who experience burns is described by the equation:

probability=1/(1+e^(-log *f* ()it))

With logit equal too:

logit = -9.584 + 0.072*(Age) + 0.090*(TBSA) + 1.804 (Presence Third Degree Burns) -2.231 (Presence Second & Third-Degree Burns) + 1.282 (Presence Diabetes) + 1.551 (Presence Inhalation Burns)

Categorical values should be treated as 1 if they are present and 0 if they are absent.

**Conclusions:**

Between 1990 and 2010 the number of people living with diabetes tripled, and the annual incidence doubled. Diabetic wound healing is characterized by excessive inflammation and reduced angiogenesis, resulting in increased risk for complications. Diabetes is an established comorbidity in burn patients, but has never been identified as a significant risk factor for mortality.

**Applicability of Research to Practice:**

Analysis of burn patients at our level 1 trauma center identified history of diabetes as a significant risk factor for mortality and suggests that the inclusion of diabetic status in future mortality models would increase their prognostic value in comparable populations.